# Medicinal Properties of *Adiantum capillus-veneris* Linn. in Traditional Medicine and Modern Phytotherapy: A Review Article

**Published:** 2018-02

**Authors:** Sahar DEHDARI, Homa HAJIMEHDIPOOR

**Affiliations:** 1. Student Research Committee, Dept. of Traditional Pharmacy, Shahid Beheshti University of Medical Sciences, Tehran, Iran; 2. Traditional Medicine and Materia Medica Research Center, Shahid Beheshti University of Medical Sciences, Tehran, Iran; 3. Dept. of Traditional Pharmacy, School of Traditional Medicine, Shahid Beheshti University of Medical Sciences, Tehran, Iran

**Keywords:** *Adiantum capillus-veneris* Linn., Maidenhair fern, Phytotherapy, Traditional medicine

## Abstract

**Background:**

*Adiantum capillus-veneris* Linn (Maidenhair fern) is an herb belonging to the family Pteridaceae. It is named as “*Pare-siavashan*” in medical and pharmaceutical textbooks of Iranian Traditional Medicine. The fronds of Maidenhair fern were mainly administrated by ancient physicians as single medicine or in combination with other plants in multi-herbal formulations for curing different diseases. Because of different chemical compositions, the herb fronds were also assessed for its numerous pharmacological effects. Therefore, the current study was done to review the traditional usage and modern pharmacological and toxicological effects of Maidenhair fern.

**Methods:**

Scientific databases and publications including Web of Science, PubMed, Scopus, Science direct, Cochrane Library, SID (for Persian papers) and medical and pharmaceutical textbooks of traditional medicine as well were searched for “*Adiantum capillus-veneris*”, “*Maidenhair fern*” and “*Pare-siavashan*” without limitation up to 2016.

**Results:**

Maidenhair fern exhibited to possess anti-diabetic, anticonvulsant, analgesic, hypocholesterolemic, goitrogenic, anti-thyroidal, antibacterial, antifungal, wound healing, antiobesity, anti hair loss, anti-asthmatic, anti-inflammatory, antidiarrheal and antispasmodic, antioxidant as well as diuretic, anti-urolithiatic and detoxifying effects in modern medicine. Ancient physicians declared some of the confirmed pharmacological effects.

**Conclusion:**

Maidenhair fern frond can be a good candidate for clinical purpose. Therefore, future researches on the other mentioned effects in traditional medicine are recommended.

## Introduction

*Adiantum capillus-veneris* Linn. (Maidenhair fern) is a tufted fern belonging to Pteridaceae family (1). The herb is widely grown in warm-temperature to tropical, with high moisture content (2). Maidenhair fern is a hardy, up to 35 cm high plant that has an aromatic fragrance with a creeping rhizome. The plant fronds are generally double-rowed, tender, glabrous, and grow up to 50 cm long. The plant almost has a glossy black petiole and is coated with hair at the base. The species has ovate to oblong-ovate leaf blade. The medicinal parts are fronds, rhizomes, and roots (3). The plant is widely distributed in various regions such as Southern Europe, Atlantic coast as far as Ireland, from the south to the southern Alpine valleys regions, from the Central to the South America, Australia and Iran (2, 3).

Maidenhair fern has been frequently used in Iranian Traditional Medicine (ITM) for different medicinal aspects. It was well known for thousands of years. Its temperament is relatively hot and dry (4–6). The plant is individually used as single medicine or in multi-herbal formulations for the treatment of different diseases (6–8). Ancient practitioners generally used fronds of Maidenhair fern as the most useful part of the herb (4, 5, 7).

The species also revealed various pharmacological effects regarding different chemical constituents such as tannins, terpenoids, flavonoids, alkaloids, and steroids (9–14). Therefore, the current research was aimed to check the traditional applications of the plant as well as pharmacological and toxicological effects in modern medicine.

## Methods

Scientific databases including Web of science, PubMed, Scopus, Science direct, Cochrane Library, SID (for Persian papers) were searched for researches focusing on the pharmacological and toxicological effects of Maidenhair fern. The utilized terms were “*Adiantum capillus-veneris*”, “*Maidenhair fern*” and “*Pare-siavashan*” without limitation up to 2016. Besides, information on the plant was collected by using medical and pharmaceutical textbooks of ITM including *Qanun fi al-Teb* (4), *Tohfat-ul-momenin* (15), *Makhzan-ol-Advieh* (7), *Zakhireh Kharazmshahi* (*Treasures of the Khwarazm Shah*) (16), *Qarabadin kabir*(17), *Qarabadin Shafaie* (18) *and Exir Azam (Great Elixir)* (19).

## Results

### Traditional applications of Maidenhair fern as single medicine

Oral application of the fronds decoction was reported to be useful for cleansing respiratory system, dyspnea, asthma, coryza and chest pain in the field of respiratory system (4). Ancient physicians also applied the eye drop in order to control the fistula lacrymalis condition (6). Oral powders of Maidenhair fern were extensively administrated for gastrointestinal disorders such as jaundice, diarrhea and abdominal cramps (7). Maidenhair fern was reported as a headache-preventing agent. It was also supposed to be effective for dissolving the kidney calculi and as a diuretic agent in oral administration (4, 7). *A. capillus-veneris* was also introduced as a powerful anti-inflammatory agent. Therefore, it was applied on fistula in the form of ointment. Persian physicians also administrated oral decoction for female genital disorders such as amenorrhea (5). The plant also helps child birth and extracting placenta with oral administration of decoction (4, 7). Maidenhair fern is a potent hair tonic that treats alopecia and helps hair growth and it is useful for dandruffs (4–7).

### Traditional applications of Maidenhair fern in multi-herbal formulations

Maidenhair fern has been extensively used in multi-herbal prescriptions for the treatment of many diseases such as respiratory, urogenital and dentistry diseases. The main multi-herbal formulations of the species along with their indications and dosage forms have been described in Table 1.

**Table 1:** The main multi-herbal formulations containing Maidenhair fern according to ITM

***Organs***	***Action(s)***	***Components***	***Dosage form***
Central nervous system	Anti Alzheimer, Brain tonic	Liquorice, Sweet Violet, Damask Rose, Lavender, Peony, Borage, Fennel, Celery, Marshmallow, Stavesacre, Assyrian Plum, Honey	Syrup (19)
	Treatment of Epilepsy, Mania and Headache	Lavender, Liquorice, Borage, Fennel, Celery, Marshmallow, Sweet Violet, Damask Rose, Stavesacre, Assyrian Plum	Syrup (18)
Dentistry	Dental analgesic	1. Liquorice, Borage flower 2. Lavender, Fumitory, Jujube, Black Nightshade aromatic water	1. Oral decoction, Mouthwash 2. Oral, decoction (19)
	Dental tonic	Frankincense, Long Aristolochi, Sweet Violet, Sandalwood	Dental Powder (17)
Hair	Anti hair loss	1. Chinaberry, Myrrh, Indian Gooseberry, Myrtle oil 2. Rockrose, Wormwood, Chio Gum 3. Myrtle, Celery seed, Radish oil	Lotion, Liniment (18,19)
Hepatic System	Hepatitis	Fenugreek, Flax, Liquorice, Marshmallow, Common Mallow, Hyssop, Fig, Stavesacre	Syrup (16)
	Jaundice	Romanwormwood, Oregano, Poley, Chamomile, Feverfew, Dill, Common Wood Sorrel, Citron	Face wash (17)
Respiratory system	Anti common cold, Antipyretic	Liquorice, Quince, Sweet Violet, Cucumber seed mucilage	Oral decoction (19)
	Antiasthmatic	Mango mucilage, Liquorice	Aromatic water (18)
	Antiasthmatic, Antitussive	Flax, Hyssop, Orris, Borage, Fennel, Liquorice, Fig, Stavesacre	Oral decoction (18)
	Antitussive	1. Liquorice, Hyssop, Sweet Violet syrup 2. Fig, Stavesacre, Liquorice, Hyssop, Orris root 3. Fennel, Celery, Liquorice, Sweet Almond kernel 4. Myrtle, Melon, Liquorice, Gum Arabic 5. Fennel, Celery, Liquorice, Bitter almond kernel, Flax mucilage	1.Oral decoction (19) 2. Oral decoction (17) 3. Pill (18) 4. Tablet (18) 5. Pill, Tablet (17)
	Pleural analgesic	Fennel, Stavesacre, Damask Rose in combination with Honey	Oral decoction (19)
	Pleural analgesic, Chest pain reliever	Pennyroyal, Anise, Liquorice, Fennel	Oral decoction (19)
	Respiratory tract tonic, Antitussive	Liquorice, Assyrian Plum, Stavesacre, Hyssop, Anise, Marshmallow, Common Mallow, Orris	Aromatic water (18)
	Treatment of Pharyngitis	Common Dodder, Chamomile, Marshmallow, Radish, Fig*,* Celery	Gargle (19)
Urogenital system	Abortive	Cretan dittany, Savin White Lupin, Pennyroyal	Oral decoction (18)
	Anti Cystolithiasis	Caltrop, Horse gram, Rusty-back, Fig	Oral decoction (19)
	Anti Nephrolithiasis	Melon, Willd Caraway, Ajwain, Galingale, Celery, Radish seed, Bitter Almond kernel	Oral decoction (16)
	Anti nephrolithiasis and Cystolithiasis	1. Horse gram, Caltrop, Melon, Fennel, Grape 2. Caltrop, Chamomile, Sweetclover, Oregano, Celery, Wild Cabbage, Dill, Marshmallow	1. Syrup (19) 2. Sitz bath (17)
	Cystitis and Nephritis	Senna, Common Polypody, Sweet Violet, Cucumber, Chicory, Plum, Jujube, Fumitory, Golden Shower	Oral decoction (19)
	Dysuria	Caltrop, Chamomile, Dill, Celery, Sweetclover, Radish, Wild Cabbage, Marshmallow, Flax, Fenugreek, Sweet Violet	Sitz bath (19)
	Emmenagogue	Cretan dittany, Fennel, Rue	Oral decoction (18)
	Emmenagogue, Abortive	Golden Shower, Cretan dittany	Oral decoction (18)
	Induction of parturition	Golden Shower, Caltrop, Melon, Savin, Cretan dittany, Marshmallow, Garden asparagus	Oral decoction (18)
	Nephralgia treatment	Wild Carrot, Caltrop, Hyssop	Oral decoction (19)
	Gonorrhea	Anise, Celery, Sweet Violet, Borage, Caltrop, Winter Cherry, Cucumber	Syrup (18)

### Pharmacological effects Anti-diabetic activity

The anti-diabetic effect of aqueous and methanol extracts of Maidenhair fern was assessed through streptozocin-induced diabetic rat model. Improvement in the fasting blood sugar exhibited that the species has very good anti-diabetic effect with low side effects. The presence of flavonoids and tannins may be responsible for the anti-diabetic effect (20). Another research exhibited significant rise in rat’s body weight and amylase enzyme and reduction in the blood glucose. The ability of the plant to gain weight is because of its repair capacity on hepato-renal damaged cell. Besides, increase in serum amylase is due to insulin-like constituents in the species which affect pancreas activity for amylase secretion (21). Besides, it was declared that the species displayed antihyperglycemic property comparable to acarbose as reference drug (22).

### Neuropharmacological activities

Neuropharmacological activities of the plant ethanoic extract were evaluated by using various methods. The plant revealed significant anticonvulsant effect through prolonging the onset of action and reduction in the period of seizures in PTZ-induced convulsion model, in addition by decrease in the time of different phases of seizure through MES-induced seizure method.

In mice forced swim assay, the species displayed depressant property by prolonging the immobility time. The species was not demonstrated remarkable skeletal muscle relaxation as well (23).

### Hypocholesterolemic effect

The hypocholesterolemic effect from water extract of *A. capillus-veneris* was evaluated by using high cholesterol diet (HCD) fed model in rats. The results exhibited potent reduction of total cholesterol (TC), LDL and VLDL serum levels with no effect on HDL level. Moreover, atherogenic index of TC/HDL was approximately normalized in rats that treated with *A. capillus-veneris* (24).

### Antiobesity effect

Aerial parts water extract of the herb exhibited phospholipase inhibitory effect through an *in vitro* model which was comparable to orlistat. Chlorogenic acid is also reported as the most responsible phytoconstituent (22).

### Goitrogenic and anti-thyroidal effects

It has been proven that after using the plant, thyroid gland weight generally decreased, although thyroid peroxidase action, antioxidant enzymes, T4 and T3 serum levels increased in animals; however TSH serum level decreased strongly (25).

### Antibacterial and antifungal activities

The antibacterial activity of Maidenhair fern against multidrug resistant (MDR) bacteria strains was evaluated through disc diffusion method. Leaves methanol extract of the species displayed maximum zone of inhibition against *Providencia, Klebsiella pneumoniae, Shigella, Vibrio cholera, Staphylococcus aureus, Proteus vulgaris* and *Salmonella typhi*. Stem methanol extract was very potent against *Escherichia coli, K. pneumonia* and *S. typhi*. Leave water extract of the species was very potent against all bacteria strains but its stem water extract revealed minimum ZI against *E. coli*, *K. pneumonia, S. typhi, Shigella, Proteus vulgaris* and *Providencia* (26). In another study, the antibacterial activities of *A. capillus-veneris* methanolic extract against *S. aureus*, *E.* *coli*, and *Helicobacter pylori* has been proven (27). In a research, crude and phenolic extracts of gametophyte and sporophyte of the plant were assessed for antibacterial properties. Antibacterial effect of gametophytic part of the plant was more significant. Gram-positive species like *Bacillus subtilis* displayed more susceptibility to both extracts (28). Moreover, the ethanolic extract of Maidenhair fern aerial parts have no antimicrobial capacities against three pathogen bacteria including *E. coli*, *Staphylococcus aureus* and *Pseudomonas aeruginosa* (29). In another research, methanolic extracts of four important *Adiantum* species including *A. capillus-veneris, A. peruvianum, A. venustum* and *A. caudatum* were evaluated for antibacterial and anti-fungal effects. Among these species *A. capillus-veneris* and *A. venustum* showed potent antibacterial properties. Besides, the antibacterial and fungal activities of leaves, stems, and roots were evaluated. Different extracts of all used parts displayed potent antibacterial and anti-fungal properties (30).

### Wound healing property

During an in vitro study, wound healing property of *A. capillus-veneris* was evaluated. The water extract of the plant improved angiogenesis significantly by using both capillary-like tubular formations and proliferation of endothelial cells. Besides, aqueous and butanol fractions revealed significant protection against damage to fibroblasts by oxygen free radicals (31). In another research, an ointment that consists of Maidenhair fern*,* Aloe vera*,* Henna and Myrrha cured wounds in diabetic rats (32).

### Anti testosterone-induced hair loss effect

The hair growth- promoting effect of ethanolic extract of Maidenhair fern was evaluated through testosterone-induced alopecia model in mice. The results revealed considerable increase in follicular density and anagen/telogen ratio (33).

### Antioxidant activity

The antioxidant capacity of ultrasonic-assisted flavonoid extract of the plant has been evaluated. *In vitro* assays were done through DPPH, scavenging capacity of superoxide anion, chelating capability of ferrous ion and reducing power tests. In vivo examination was done by using acute mice liver injury experiment. The results exhibited more potent antioxidant activity of the species than some synthetic antioxidants such as BHT, EDTA, and ascorbic acid. *In vivo* evaluation displayed significant decrease in superoxide dismutase (SOD), catalase (CAT) and glutathione (GSH) levels and notable increase in malondialdehyde (MDA) levels (34). In another in vitro investigation, ethanolic extract of *A. capillus-veneris* leaves has assessed against hydrogen peroxide-induced oxidative damage in peripheral blood lymphocytes. The results demonstrated inhibition of lipid peroxidation and increase in the level of antioxidant enzymes including SOD, CAT, Gpx and glutathione content (35). During an in vitro study, antioxidant activity of the plant essential oil was confirmed through DPPH assay. Antioxidant property of the essential oil is because of phytoconstituents such as carvone, carvacrol, and thymol (9). In addition, *A. capillus-veneris* and *M. punctatum* were compared and reported that the rises in the malondialdehyde levels and antioxidant enzymes including superoxide dismutase and glutathione peroxidase in *M. punctatum* were more potent (36).

### Urinary tract effect

The efficacy of *A. capillus-veneris* water extract was assessed on urinary tract. The result exerted inhibition effect on all tested bacterial species in this experiment. Systemic *Candida albicans* infection model was employed in mice to assess the protective activity of the plant. It also reduced the colony-forming units (CFU) of *C. albicans* in the spleen and improved the renal pathological characteristics. Besides, it displayed double effects on diuresis activity. The low dosage generally raised the urinary output and high dose significantly reduced the urinary output. *A. capillus-veneris* can be used for treatment of urinary tract infection (UTI) (37). In another research, hydroalcoholic extract of *A. capillus-veneris* was evaluated for anti-calcium oxalate urolithiasic property by male rats. The results revealed significant decrease in the number of crystals and reduction in the serum level of calcium, phosphorous and blood urea (38). They also confirmed this effect during an in vitro study. The plant restrained the crystallization, crystal aggregation, and reduction in the number and the sizes of crystals (39).

### Anti-inflammatory activity

The ethyl acetate fraction of the plant ethanolic extract has displayed significant anti-inflammatory activity related to the inhibition of NO release and reducing in TNF-α level. Triterpenes may play chief role in the anti-inflammatory property of the plant (40). Moreover, during an in vitro study, the anti-inflammatory activity of the plant ethanolic extract was assessed through lipopolysaccharide-induced prostaglandin E2 generation in RAW 264.7 macrophage and interleukin 6 and tumor necrosis factor generation in the human monocyte model. The plant notable anti-inflammatory property is because of suppressing effect on nuclear factor kappa B activation, due to inhibitory effect on the production of inflammatory cytokines (41). In another investigation, two triterpenoids including 30-normethyl fernen-22-one and 4-α- hydroxyfilican-3-on that isolated from fronds ethanolic extract presented potent anti-inflammatory activity by using carrageenan-induced hind paw edema test in rat (12).

### Analgesic and antinociceptive activities

The analgesic effect of the ethyl acetate fraction of the ethanolic extract from Maidenhair fern has been confirmed through tail-flick and writhing methods (40). Similar investigation confirmed powerful analgesic effect of Maidenhair fern through hot plate and tail immersion tests in mice (23). In addition, 4-α- hydroxyfilican-3-on that isolated from ethanolic extract of the plant showed significant anti nociceptive activity in writhing test (12).

### Antidiarrheal and antispasmodic activities

In a research, the crude extract of *A. capillus-veneris* dried leaves was evaluated for antidiarrheal and antispasmodic capacities. Antidiarrheal effect was proved through castor oil-induced diarrhea in mice model. Furthermore, inhibitory effect on K+-induced contraction was seen in isolated rabbit jejunum preparation that confirmed the antispasmodic activity of the plant (42).

### Anti-asthmatic activity

The anti-asthmatic effect of the ethanolic extract from *A. capillus-veneris* leaves was confirmed through histamine aerosol-induced asthma in guinea pig. It should be noted that the herb has been traditionally used as anti-asthmatic agent (43).

### Detoxification activity

Crude extract of Maidenhair fern has demonstrated powerful protection through bisphenol A-induced reproductive system toxicity in rats (44). Besides, the ethanol extract of the plant at 500 mg/kg doses after 14 d therapy, revealed remarkable nephroprotective activity against cisplatin-induced nephropathy (45).

#### Toxicity and adverse reactions:

During an in vitro study, the effects of the ethanolic and aqueous extracts of maidenhair fern on aryl hydrocarbon hydroxylase (AHH) and epoxide hydrolase (EH) enzyme activities, which are responsible for accelerating conversion of carcinogenic compounds like poly aromatic hydrocarbons to active components, were assessed. Both plant extracts revealed no inhibitory effect on AHH and EH enzymes (46).

Toxicity study of Maidenhair fern ethanolic extract was done in rat. The results exhibited behavioral reactions in rats at the dose of 300 mg/kg. But no mortality was seen after 72 h (40).

Crude extract of Maidenhair fern at 1, 3 and 7 g/kg was administrated orally in mice. No sign of acute toxicity including seizure, piloerection and restlessness were reported after 6 h. Besides, after 24 h no mortality was seen in mice (42).

Acute oral toxicity studies of the aqueous and methanolic extracts were done in rat. Acute dosage was 2000 mg/kg as single dose. After first 30 min, 4 h and 24 h after administration, main changes in behavior and death were evaluated. Both extracts exhibited no major changes in behavior and no lethality as well (20).

For evaluation of acute toxicity, ethanol extracts of the species at the oral doses of 1000 and 2000 mg/kg were administrated to mice. After 24 h no sign of behavioral changes or mortality was seen (25). However, the plant should not be used during lactation period because of no available data. The plant is also contraindicated in pregnant women (3).

According to ITM, Maidenhair fern could damage spleen, so the herb should not be used in susceptible patients (7).

### Dosage

In modern medicine, 1.5 g of powdered drug is used as tea bag daily (3). According to ITM, in the form of decoction 20 g of the fronds is accepted (7).

### Commercial products

In the form of topical solution, it is used as hair tonic three times daily. In addition, the plant is used in anti-dandruff shampoos and moisturizing creams.

## Discussion

**Fig. 1:**
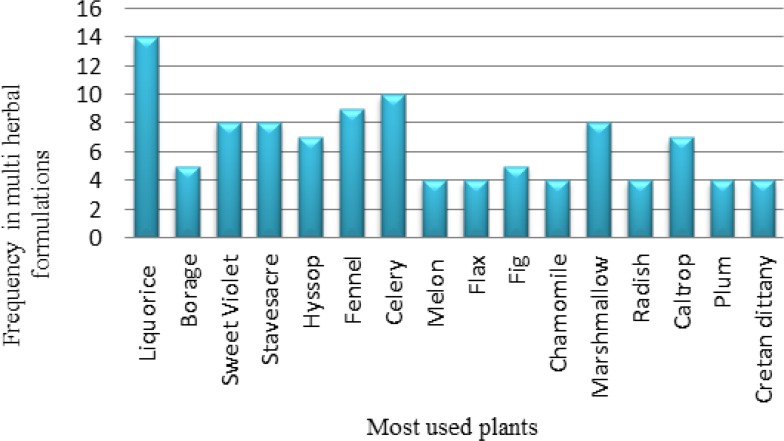
The most used plants in combination with Maidenhair fern based on ITM

Many pharmacological effects including anti-diabetic, anti-obesity, anticonvulsant, analgesic, hypocholesterolemic, goitrogenic, anti-thyroidal, antibacterial, antifungal, wound healing, anti-hair loss, anti-asthmatic, anti-inflammatory, antidiarrheal and antispasmodic, antioxidant as well as diuretic, anti-urolithiasis, and detoxifying properties are ascribed to *A. capillus-veneris*. According to ITM, Maidenhair fern has been prescribed as single medicine or in poly, herbal formulations for the treatment of many diseases and among them respiratory and urogenital diseases were the most important one. The most used plants in combination with Maidenhair fern in traditional multi-herbal formulations have been revealed in Fig. 1, and among them liquorice, celery, fennel, sweet violet, Stavesacre, marshmallow, caltrop, and hyssop were the most cited plants.

The plants used with Maidenhair fern in traditional multi-herbal formulations demonstrated related biological effects individually.

For example liquorice, sweet violet and marshmallow were studied for antitussive effect (47–50). Liquorice and caltrop established antispasmodic activity (47, 51). Liquorice, celery, and caltrop showed diuretic property (47, 51, 52). Liquorice has been introduced as anticonvulsant agent as well (53). Moreover, anti-inflammatory effect of liquorice, caltrop, hyssop, fennel, celery has been confirmed (47, 51, 52, 54, 55). Sweet violet and hyssop have been considered as brain tonic and anti-asthmatic herbs (48, 54). Caltrop has shown analgesic and anti-urolithiasis effects (51). No related research was found about biological activities of stavesacre.

**Fig. 2:**
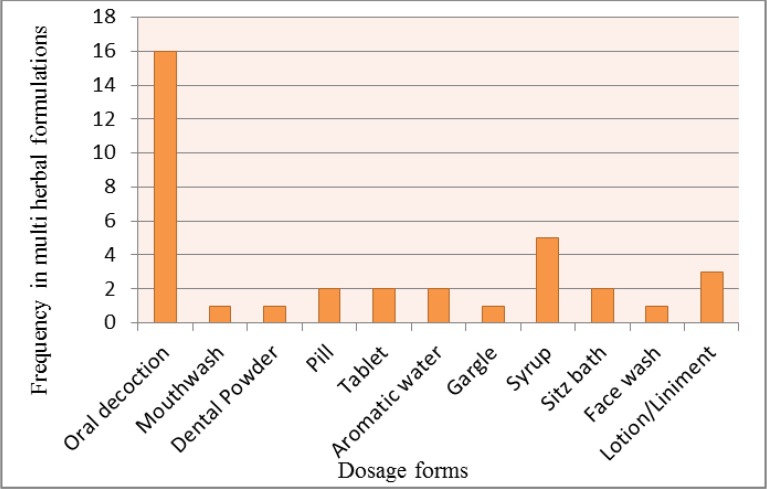
Dosage forms frequencies of Maidenhair fern multi-herbal formulations based on ITM

Among various dosage forms were utilized in poly herbal formulations of Maidenhair fern, oral decoction, syrup and lotion/liniment were the most used dosage forms (Fig. 2). According to traditional usages and pharmacological effects of the plant, some effects are similar in traditional and modern medicines.

These therapeutic similarities are including anticonvulsant, antispasmodic, diuretic, anti-lithiastic, anti-hair loss, anti-inflammatory and analgesic properties. In a mini review, some pharmacologic effects including antioxidant, anti-inflammatory, diuretic, antimicrobial, antidiabetic and antinociceptive as well as some medicinal properties of Maidenhair fern in ITM were reviewed. However, the mentioned activities were very brief compared to the recent work (56).

## Conclusion

Maidenhair fern is a plant with important effects in both traditional and modern medicines. Eight herbal medicines including liquorice, celery, fennel, sweet violet, stavesacre, marshmallow, caltrop, and hyssop can be used with maidenhair fern in multi herbal formulations maybe because of their synergistic effects. Some of the confirmed pharmacological effects in modern medicine including anticonvulsant, antispasmodic, diuretic, antilithiastic, anti-hairloss, anti-inflammatory and analgesic effects were declared by ancient physicians as well. Therefore, future researches on the other mentioned effects in ITM are recommended. In addition, with reference to these various pharmacological properties, *A. capillus-veneris* frond can be a good candidate for clinical purpose. However, in spite of different in vitro and in vivo researches, lack of comprehensive clinical trials focused on considered activities are remaining to establish the traditional information.

## Ethical considerations

Ethical issues (Including plagiarism, informed consent, misconduct, data fabrication and/or falsification, double publication and/or submission, redundancy, etc.) have been completely observed by the authors.
